# Impact of Huanglongbing Pathogen Infection on the Amino Acid Composition in Both Citrus Plants and the Asian Citrus Psyllid

**DOI:** 10.3389/fphys.2021.777908

**Published:** 2021-12-10

**Authors:** Li-He Zhang, Su-Li Ren, Zheng-Qin Su, Pei-Ping Xu, Da Ou, Li-Jun Wang, Wen Sang, Bao-Li Qiu

**Affiliations:** ^1^Chongqing Key Laboratory of Vector Insects, College of Life Sciences, Chongqing Normal University, Chongqing, China; ^2^Guangdong Laboratory for Lingnan Modern Agriculture, Guangzhou, China; ^3^Guangdong Laboratory of Lingnan Modern Agriculture, Genome Analysis Laboratory of the Ministry of Agriculture and Rural Affairs, Agricultural Genomics Institute at Shenzhen, Chinese Academy of Agricultural Sciences, Shenzhen, China; ^4^Airport Management College, Guangzhou Civil Aviation College, Guangzhou, China

**Keywords:** Asian citrus psyllid, citrus huanglongbing, phloem sap, amino acids, honeydew

## Abstract

The Asian citrus psyllid (ACP) *Diaphorina citri* is the main vector of the pathogen *Candidatus* Liberibacter asiaticus (*C*Las), which is the causal agent of citrus Huanglongbing disease. Feeding by both ACP nymphs and adults on host plants allows them to obtain nutrition. Therefore, the nutritional content within the plant phloem is of much importance for the development and reproduction of ACP. The infection by pathogenic microbiomes may affect the amino acid contents of their host plants and then indirectly affect the biology of sap-feeding insects. In this study, we investigated the amino acid contents and their proportions in both *C*Las-infected and *C*Las-free citrus plants, ACP adults, and also in honeydew produced by ACP nymphs. Results showed that infection by *C*Las had a large impact on the amino acid species and proportion in all the tested target plants, ACP adults, and in the honeydew of ACP nymphs. The content of total amino acids in *C*Las-infected citrus was much higher than that of *C*Las-free citrus. However, *C*Las infection significantly reduced the proportion of essential amino acids (EAAs) in these plants. When feeding on *C*Las-infected citrus plants, ACP adults absorbed less total amino acids than those adults feeding on healthy plants, but the proportion of EAAs was significantly higher when they fed on *C*Las-infected citrus plants. The proportion of EAAs also significantly increased in the honeydew secreted by ACP nymphs that fed on *C*Las-infected citrus plants. However, EAA detection in the honeydew of ACP nymphs indicated that the utilization rate of EAAs by *C*Las positive ACP nymphs was reduced. Our study has revealed that *C*Las infection significantly affects the contents, proportion, and utilization efficiency of different amino acids in citrus plants, ACP adults, and nymphs, leading to a developmental pattern of ACP that is more conducive to *C*Las transmission.

## Introduction

Huanglongbing (HLB), commonly known as citrus greening disease, is caused by three species of the fastidious, phloem-residing, gram-negative bacteria “*Candidatus* Liberibacter asiaticus” (*C*Las; Bové, 2006), “*Ca.* L. africanus” (*C*Laf; Garnier et al., 2000), and “*Ca.* L. americanus” (*C*Lam; Texeira et al., 2005). It is one of the most economically destructive diseases of the citrus industry worldwide (Wang et al., 2016). At present, there is no established cure for this century-old disease. HLB, whose name in Chinese means “yellow dragon disease,” was first reported from southern China in 1919 but is thought to most likely have originated in Taiwan Island in the 1870s. At present, it is now known to occur in approximately 40 different countries across Asia, Africa, Oceania, North America, and South America (Bové, 2006; Belasque et al., 2010).

The HLB pathogen resides in the phloem of host plants and causes a systemic disease (Jagoueix et al., 1994), for example, it has caused crippling diseases denoted “greening” in South Africa, “mottle-leaf” in the Philippines, “dieback” in India, and “vein phloem degeneration” in Indonesia (Bové, 2006). The HLB-infected trees display a blotchy mottle condition of the leaves that results in the development of yellow shoots, which is the early and typical characteristic symptom of the disease. The trees then gradually become stunted, bearing only a few small-sized and deformed (lop-sided) fruits, which are poorly colored (greening). The fruit coloration starts at the peduncular end (color inversion) and results in fruit loss and eventual tree death (Roistacher, 1996; Bové, 2006).

The amino acid content in the phloem sap of host plants is the important nutritional component for the successful development and reproduction of piercing-sucking insects such as whitefly, psyllids, aphids, white wax scale, plant hopper, and leafhoppers; these insects take amino acids directly from leaf liquid instead of proteins (Ding, 1990; Zhao et al., 2001). It is thought that pathogenic microbiomes may affect the amino acid content of host plants and, as a result, indirectly affect the behavior of insects feeding on the plants. For example, it has been found that cassava plants infected with East African cassava mosaic virus (EACMV) can significantly increase the population growth of the whitefly *Bemisia tabaci*. It was deduced that this virus perhaps improves the contents of Glutamine, Aspartic acid (Asp), Tryptophan (Trp), and Tyrosine (Tyr) in the phloem sap of cassava plants (Colvin et al., 2006). Jiu et al. (2007) found that in comparison with its performance on healthy tobacco, *B. tabaci* showed a significantly increased fecundity and longevity through feeding on tobacco plants infected with two begomoviruses. Other studies have also shown that the aphid *Aphis gossypii* feeding on *Zucchini yellow mosaic virus*-infected pumpkin can increase their fecundity and longevity. Thus, it is speculated that the increase of fecundity and longevity may be closely related to the higher amino acid concentration in the phloem sap of pathogen-infected host plants (Blua et al., 1994; Stout et al., 2006).

The Asian citrus psyllid (ACP), *Diaphorina citri* Kuwayama (Hemiptera, Liviidae), is the main vector of bacteria in the genus *Liberibacter*, which are the causal agents of HLB (Tsai et al., 1988; Onagbola et al., 2011). Amino acids required by most insects are obtained from food proteins. However, some piercing-sucking insects, such as white wax scale, plant hopper, and leafhopper, directly take amino acids from the host leaf liquid instead of those *via* proteins (Ding, 1990; Zhao et al., 2001). ACP immatures and adults need to assimilate nutrition from host plants for their successful growth and development. There are studies showing that aphids increase their own fecundity after feeding on susceptible host plants. It could be speculated that the increase in fecundity may be closely related to an increase of some amino acids in the phloem sap of the susceptible host plant (Holopainen et al., 1997; Sandström, 2000; Walter and Difonzo, 2014). Ren et al. (2016) revealed that the infection of *C*Las both in citrus shoots and ACP produced a population increase of ACP. However, the mechanisms underlying the nutrition-related interactions among *C*Las pathogen, host plants (especially with respect to amino acid nutrition), and ACP are not completely understood.

Therefore, it is necessary to explore the link between the free amino acids (FAAs), essential amino acids (EAAs) required by ACP, and the phloem amino acid composition of host plants after infection with *C*Las pathogen. In this study, we compared the changes of total FAA content and their proportions in different treatments (*C*Las-infected and *C*Las-free citrus plants, *C*Las-positive and -negative ACP, and *C*Las-positive and -negative honeydew). The purpose of the study was to clarify the nutritional relationship between the HLB pathogen, citrus host plants, and the vector insect ACP. From the perspective of amino acid utilization, the effect of the HLB pathogen on the absorption and utilization of amino acids by the ACP was specifically considered.

## Materials and Methods

### Host Plants

The HLB-free and HLB-infected citrus trees “Shatangju” (*Citrus flamea* Hort. ex Tseng shiyueju) were first selected from two different citrus groves in Zhaoqing, Guangdong, China in November 2017. These trees were completely removed, reinstated, watered, and fertilized as required in the experimental field site of the campus of South China Agricultural University (SCAU), Guangzhou, China. Nested quantitative PCR (qPCR) detection was performed periodically to detect the *C*Las infection in these citrus plants using the method described by Coy et al. (2014). In brief, the nested PCR increases the specificity and sensitivity of detection. Nested PCR uses two pairs of PCR primers to amplify the complete fragment. In the first step, the target DNA is combined with the first pair of common primers. In the next step, the second set of nested primers (inside the first PCR amplification fragment) is used to perform the second PCR on the products amplified by the first round of PCR. The first pair of primers used for *C*Las detection was 5′-AAGGAGGTGATCCAGCCGC-3′, 27F: 5′-AGAGTTTGATCATGGCTCAG-3′ in this study. The PCR procedure entailed pre-denaturation at 94°C for 5 min then followed by 20 cycles of 94°C for 30 s, 50°C for 30 s, and 72°C for 1 min, and finally a 4-min extension period at 72°C. The total system for the first round of PCR amplification is 25 μl, including 2.5 μl PCR buffer, 2.5 μl dNTPs, 0.5 μl upstream and downstream primers, respectively, 0.4 μl Taq enzyme, 1 μl DNA template, and 17.6 μl ddH2O. The second pair of primers used for *C*Las detection was OI1: 5′-GCGCGTATGCAATACGAGCGGCA-3′ and OI2c: 5′-GCCTCGCGACTTCGCAACCCAT-3′. The PCR procedure entailed pre-denaturation at 96°C for 1 min then followed by 35 cycles of 94°C for 30 s, 55°C for 30 s, and 72°C for 1 min, and finally a 4-min extension period at 72°C. The total system for the second round of PCR amplification is 25 μl, including 2.5 μl PCR buffer, 2.5 μl dNTPs, 0.5 μl upstream and downstream primers, respectively, 0.4 μl Taq enzyme, 2 μl DNA template, and 16.6 μl ddH2O.

### Insects

The *C*Las-negative colony of the ACP was originally collected from HLB-free plants of *Murraya paniculata* (L.) in field sites belonging to SCAU in 2017. Following collection, the ACP was tested for *C*Las to ensure that it was a *C*Las-negative population. The ACP population was continuously reared on the young shoots of healthy citrus plants. The *C*Las-positive colony of ACP was set up by allowing ACP adults to reproduce a new generation on *C*Las-infected citrus shoots. Similarly, the infection status of ACP adults was monitored periodically by nested-qPCR according to the description of Coy et al. (2014). Both the *C*Las-positive and *C*Las-negative ACP populations were reared for 10 generations on these HLB-free and HLB-infected citrus trees, respectively, before being used in experimental trials.

### Phloem Sap Collection

To collect the phloem sap exudates of a citrus plant, *C*Las-infected and *C*Las-free citrus plants were watered and fertilized and then pruned to encourage new shoot growth. Three shoots with 2–3 pieces of unfolded leaves of each kind of plant were quickly cut with an operating knife blade and inserted into a centrifuge tube with 2 ml of ethylenediaminetetraacetic acid (EDTA) solution (0.02 mol/L, pH 7.0). The samples were then placed in the dark at 26°C, RH > 90% for 12 h. Following this, the citrus shoots were removed, and the solution was diluted to 4 ml with EDTA and subsequently filtered with a syringe filter and conserved at −20°C for experimental use (Su et al., 2015; Guo et al., 2019).

### Asian Citrus Psyllid Honeydew Collection

To collect the honeydew, a dustpan-shaped box made of tinfoil was fixed under the tender branches where ACP nymphs were abundantly fed. As a result, the honeydew secreted by the ACP nymphs fell onto the tinfoil container. The whole branch and tinfoil box were covered with nylon bags to avoid the interference of other insects, such as ants. Following 1 week, the honeydew on the dustpan-shaped tinfoil box was gently collected into 1.5-ml centrifugal tubes and carefully weighed before being stored at −20°C for experimental trials.

### Amino Acid Analyses

#### Free Amino Acids From Phloem Sap and Asian Citrus Psyllid Nymph Honeydew

The collected phloem sap exudates of *C*Las-infected and *C*Las-free citrus trees, and the collected ACP nymph honeydew was first diluted with 1 ml 8% sulfosalicylic acid. Then, 100 μl of diluent and FAA standard solutions were put into 1.5-ml centrifugal tubes, respectively. Then, the same volume of ninhydrin and its buffer were added into each centrifugal tube and boiled for 5 min. The color of phloem sap exudate and honeydew solutions was observed and diluted with 6% sulfosalicylic acid as required until their color was close to the FAA standard solution. Both solutions were then filtered through a 0.45-μm filter. The FAA contents were analyzed using an amino acid analyzer S433 (Sykam, Munich, Germany), and all the analyses were performed with three biological replicates.

#### Free Amino Acids From Asian Citrus Psyllid Adults

The examination of FAAs was performed as described by Pan et al. (2013). In brief, approximately 80 ACP adults (7 days old) were collected from HLB-free and HLB-infected citrus trees, respectively. They were first ground in liquid nitrogen, dissolved in 1.5 ml ddH_2_O, with the solutions then shaken for 2 min on a vortex shaker, and centrifuged at 14,000 rpm for 10 min. Following this, the supernatant was successively mixed with n-hexane, sulfosalicylic acid, and sodium citrate buffer solution, centrifuged at 10,000 rpm for 10 min between each two mixture treatments, before finally being passed through a 0.45-μm filter. As with the FAA analysis in the phloem sap exudate, the FAA contents in ACP adults were analyzed using an amino acid analyzer S433 (Sykam, Munich, Germany), and all the analyses were performed with three biological replicates.

### Statistical Analyses

The FAAs were determined by the automatic amino acid analyzer. The percentage of the various amino acids was calculated by the following formula: the respective amino acid content/the total amino acid content × 100%. The contents of total amino acids and the percentage of EAAs and non-essential amino acids (NEAAs) were analyzed using SPSS 19.0 (for Windows; SPSS, Chicago, IL, United States), and the differences were compared using the *t*-test at a significant level of *P* < 0.05.

## Results

### The Contents of Free Amino Acids in the Phloem Sap of *Candidatus* Liberibacter Asiaticus-Infected and *Candidatus* Liberibacter Asiaticus-Free Citrus

**FIGURE 1:**
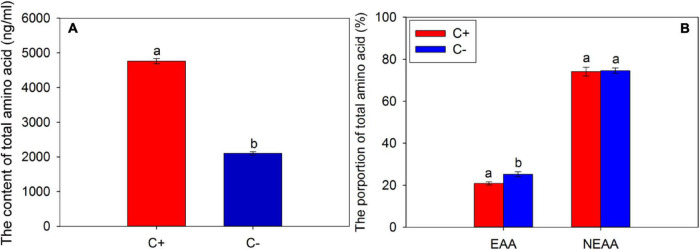
The contents of a total amino acid **(A)** and the proportions of essential amino acids (EAAs) and non-essential amino acids (NEAAs) **(B)** in the phloem sap of citrus plants. “C+” and “C−” represent *Candidatus* Liberibacter asiaticus (*C*Las)-infected and *C*Las-free citrus plants, respectively; EAA, essential amino acid; NEAA, non-essential amino acid. Values are represented as mean ± SE, and different letters over the bars mean significant differences between groups at *P* < 0.05.

According to the results of the determination of FAAs in the phloem sap, the contents of total amino acids in the phloem of *C*Las-infected citrus were significantly higher than that of *C*Las-free citrus (*t* = 42.401, df = 2, *P* < 0.001) (Figure 1A). In this study, we selected the same species of amino acids detected in the phloem sap of *C*Las-infected and *C*Las-free citrus plants for analysis and found that the proportion of EAAs in the *C*Las-infected citrus plants was significantly lower than that of the *C*Las-free citrus plants (*t* = −18.395, df = 2, *P* = 0.003) (Figure 1B), which means the infection of *C*Las significantly reduced the proportion of EAAs in the *C*Las-infected citrus plants (*t* = −18.395, df = 2, *P* = 0.003), but no significant difference was found between the NEAA proportions of *C*Las infection and *C*Las-free citrus plants (*t* = −2.014, df = 2, *P* = 0.182).

### Free Amino Acid Composition in the Phloem Sap of Citrus Plants

**FIGURE 2:**
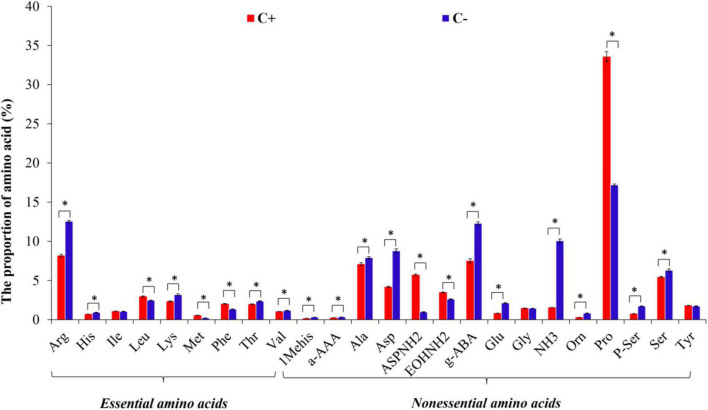
The species of free amino acids (FAAs) in the phloem sap of citrus plants. “C+” and “C−” represent *C*Las-infected and *C*Las-free citrus plants, respectively. Values are represented as mean ± SE, and * over the bars means significant differences between groups at *P* < 0.05.

A total of 24 FAAs were detected in both *C*Las-infected and *C*Las-free citrus plants, with NEAAs being the main amino acid components in the phloem sap of citrus plants. However, *C*Las infection significantly affected the contents of 21 amino acids except Leucine (Leu), Glycine (Gly), and Tyr. For example, *C*Las infection greatly increased the mass percentage of Asparagine (AspNH_2_) and Proline (Pro) but decreased the mass percentage of Arginine (Arg), Alanine (Ala), Asp, γ-aminobutyric acid (g-ABA), Ammonia (NH_3_), Phosphatidylserine (P-ser), and Serine (Ser) in the phloem sap of *C*Las-infected citrus plants (Figure 2). The HLB pathogen, therefore, has a significant influence on the proportion of most components of EAAs and NEAAs in the host plant (*P* < 0.05).

### The Contents of Free Amino Acids in Asian Citrus Psyllid Adults

**FIGURE 3:**
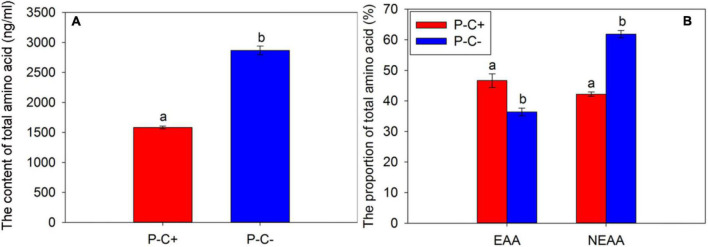
The content of total amino acids **(A)** and the proportions of EAAs and NEAAs **(B)** in the Asian citrus psyllid (ACP) adults feeding on *C*Las-infected and *C*Las-free citrus plants. “P+” and “P−” represent *C*Las positive and negative psyllids, respectively, and “C+” and “C−” represent *C*Las-infected and *C*Las-free citrus pants, respectively. EAA, essential amino acids; NEAAs, non-essential amino acids. Values are represented as mean ± SE, and different letters over the bars mean significant differences between groups at *P* < 0.05.

The *C*Las infection greatly impacted the contents of total FAAs of ACP adults. Compared with the ACP adults that fed on *C*Las-free citrus plants, the contents of total amino acids of ACP adults that fed on *C*Las-infected citrus decreased significantly (Figure 3A) (*t* = −18.395, df = 2, *P* = 0.003). In this study, we selected the same species of amino acids detected in the *C*Las-positive and *C*Las-negative ACP populations for analysis and found that the EAA proportion of the ACP adults feeding on *C*Las-infected citrus plants was significantly higher than that of the ACP adults feeding on *C*Las-free citrus plants (*t* = 24.861, df = 2, *P* = 0.002), while the proportion of NEAAs showed the opposite trend (*t* = −94.353, df = 2, *P* < 0.001) (Figure 3B). These results may reflect that the ACP adults that fed on *C*Las-infected citrus plants absorbed more EAAs than those ACP adults feeding on *C*Las-free citrus plants.

### Free Amino Acid Composition in the Asian Citrus Psyllid Adults

**FIGURE 4:**
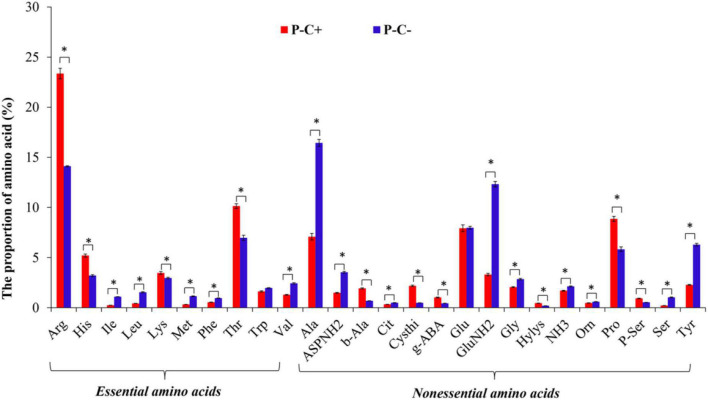
The species of FAAs in the ACP adults feeding on *C*Las-infected and *C*Las-free citrus plants. “P+” and “P–” represent *C*Las positive and negative psyllids, respectively, and “C+” and “C–” represent *C*Las-infected and *C*Las-free citrus plants, respectively. Values are represented as mean ± SE, and * over the bars means significant differences between groups at *P* < 0.05.

The same total of 26 FAAs was identified in the ACP adults that fed on both *C*Las-free and *C*Las-infected citrus plants. *C*Las infection significantly influenced the proportion of the EAAs especially Arg, Histidine (His), and Threonine (Thr), and the NEAAs, in particular, Ala, L-isoglutamine (GluNH_2_), Pro, and Tyr (*P <* 0.05) (Figure 4).

### Free Amino Acid Composition in the Honeydew of Asian Citrus Psyllid Nymphs

**FIGURE 5:**
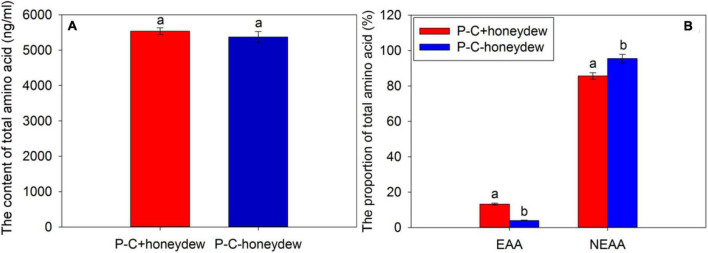
The contents of total amino acids **(A)** and the proportions of EAAs and NEAAs **(B)** in honeydew excreted by ACP nymphs feeding on *C*Las-infected and *C*Las-free citrus plants. “P+” and “P–” represent *C*Las positive and negative psyllids, respectively, and “C+” and “C–” represent *C*Las-infected and *C*Las-free citrus plants, respectively. Values are represented as mean ± SE, and different letters over the bars mean significant differences between groups at *P* < 0.05.

The analysis of FAAs in the honeydew of ACP nymphs revealed that the contents of total amino acids in the honeydew secreted by the ACP nymphs from *C*Las-infected citrus plants were higher than that of ACP nymphs from *C*Las-free citrus plants, but the difference was not significant (*t* = 0.790, df = 2, *P* = 0.512) (Figure 5A). In this study, we selected the same species of amino acids detected in the honeydew of *C*Las-positive and *C*Las-negative ACP populations for analysis and found that the *C*Las infection significantly affected the proportions of EAAs and NEAAs. Compared with the honeydew from ACP nymphs feeding on *C*Las-free citrus plants, the proportion of EAAs increased significantly (*t* = 61.518, df = 2, *P* < 0.001), while the proportion of NEAAs decreased significantly in the honeydew of ACP nymphs feeding on *C*Las-infected citrus plants (*t* = −6.654, df = 2, *P* = 0.022) (Figure 5B). Results indicated that the efficiency of EAA absorption for ACP nymphs was increased, but the utilization was reduced when they fed on *C*Las-infected citrus plants.

### Free Amino Acid Species in the Honeydew of Asian Citrus Psyllid Nymphs

**FIGURE 6:**
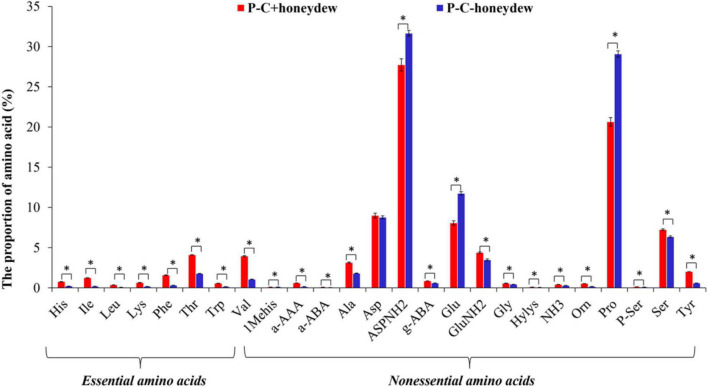
The species of FAAs in honeydew excreted by ACP nymphs feeding on *C*Las-infected and *C*Las-free citrus plants. “P+” and “P–” represent *C*Las positive and negative psyllids, respectively, and “C+” and “C–” represent *C*Las-infected and *C*Las-free citrus plants, respectively. Values are represented as mean ± SE, and * over the bars means significant differences between groups at *P* < 0.05.

Similar to the FAAs in ACP nymphs, the same total of 25 species of FAAs was identified in the honeydew secreted by the ACP nymphs feeding on either *C*Las-free or *C*Las-infected citrus plants. The *C*Las infection significantly affected the EAAs proportion in ACP honeydew, mainly increasing the proportions of the EAAs such as His, Isoleucine (Ile), Leu, Lysine (Lys), Phenylalanine (Phe), Thr, Trp, and Valine (Val) while decreasing the proportions of NEAAs such as ASPNH_2_, Glutamic acid (Glu), and Pro in contrast to the proportions of other NEAAs, which were increased significantly (*P* < 0.05) (Figure 6).

## Discussion

Plants are frequently damaged by insect feeding and their associated vectored pathogens. These insect-vectored pathogens can alter numerous host plant factors, such as odors, visual, tactile, induced defense, sugars, FAA, and secondary metabolites (Bosque-Pérez and Eigenbrode, 2011; Casteel et al., 2014; Mauck et al., 2014a,b). ACP is a hemipteran insect with a piercing-sucking mouthpart that takes the phloem exudates of its host plant as its main food and nutrition source. In plant phloem exudates, the composition and content of amino acids are complex with FAAs being the main component (Sandström, 2000; Karley et al., 2002). In some early studies, a positive correlation was observed between the number of whitefly *B. tabaci* individuals and the amino acid content of their host plant (Crafts-Brandner, 2002). A subsequent study revealed that the concentrations of Ser, Ala, Pro, Phe, Glu, Asp, Arg, and Trp in the host plant played an important role in the survival rate of *B. tabaci* (Thompson, 2006). In our study, *C*Las infection significantly altered the content of total FAAs and the proportions of EAAs and NEAAs in the phloem sap of citrus plants, which is consistent with earlier studies on other interactions between plants and pathogens (Casteel et al., 2014; Su et al., 2015). In more detail, we found that *C*Las infection significantly reduced the FAA contents in the phloem of host plants, especially Ser, Ala, Glu, and Arg amino acids; these changes may affect the survival rate of ACP nymphs, supporting the previous findings of Ren et al. (2016).

In plant-pathogen-insect vector systems, the pathogen can directly or indirectly affect the insect behavior through the alteration of host plant physiology (Colvin et al., 2006; Stout et al., 2006; Belliure et al., 2010). Many insect herbivores prefer to select virus-infected plants because virus-infected plants often display better nutritional quality, more efficient absorption of nutrients, or repressed anti-herbivore defenses (Mauck et al., 2012; Wang, 2012; Ángeles-López et al., 2016). Our study further revealed that *C*Las infection increased the proportion of EAAs in ACP adults. It has been reported that the HLB pathogen lacks a complete aerobic respiratory pathway, so it needs an insect or plant host to provide it with EAAs and energy sources (Hijaz and Killiny, 2014). Further studies indicated that the HLB pathogen may manipulate the synthesis pathway of FAAs in ACP to supplement the intermediate products lacking in the tricarboxylic acid cycle (Killiny, 2018; Killiny et al., 2018). All these findings assist in the understanding of why the proportion of EAAs was higher than that of NEAAs in the ACP adults feeding on *C*Las-infected citrus plants.

It is well known that many amino acids, especially the EAAs obtained from diets, cannot be synthesized in insects but are necessary for their development and reproduction (Hansen and Moran, 2011; Boudko, 2012), for example, as phloem-feeders, aphids, and whiteflies absorb a diet that contains fairly high levels of FAAs (Buchanan et al., 2000). Stout et al. (2006) studied nutrition-related interactions between aphids and virus-infected plants where they found that the performance of aphids is often related to the nutritional quality of phloem sap. *Rhopalosiphum padi* and *Sitobion avenae* aphids showed both positive preference to and increased fecundities on cereal plants infected with *Barley yellow dwarf virus* compared to those on healthy cereals (Ferereset et al., 1989; Jiménez-Martínez et al., 2004a,b).

The development and reproduction of insects are not only correlated with the quality of diet but also related to their own utilization efficiency of nutrition (Montllor, 1989). The results of our study showed that *C*Las infection remarkably increased the mass proportions of Thr, His, Arg, and Pro in the ACP adults that fed on *C*Las-infected citrus plants, which indicated that the digestion and utilization efficiency of Pro by ACP adults was improved by *C*Las infection. Pro is the energy source of insects, and the oxidation of Pro through the Pro-Ala cycle has been found to be the major source of fuel for flight muscles in some insects (Crafts-Brandner, 2002). ACP adults from *C*Las-infected citrus have been found to be more active in migration between different citrus trees. The high efficiency of energy utilization of Pro may explain the energy source for frequent migration of ACP, and this stimulated dispersion and flight ability will facilitate the spread of *C*Las (Martini et al., 2015; Stelinski, 2019).

The content of the total amino acids was not significantly different between the honeydew secreted by the ACP nymphs feeding on *C*Las-infected and *C*Las-free citrus plants, but there were significant differences between the proportion and components of EAAs between the two types of honeydew. Taking this above finding, that is, *C*Las infection increased the proportion of EAAs in the honeydew of ACP nymphs, we proposed that the EAA utilization efficiency of those ACP nymphs feeding on *C*Las-infected citrus was much lower than those ACP nymphs feeding on *C*Las-free citrus plants.

Mann et al. (2018) studied the effects of *C*Las infection on the development of midgut epithelial cells of *D. citri*. In this study, the midgut epithelial cells of nymphs exhibited a lower level of karyorrhexis in their early stage when they were infected with *C*Las. The midgut of an insect is the main site for the secretion of digestive enzymes, food digestion, and nutrient absorption. The efficiency decrease in the EAA absorption and utilization of the ACP nymphs feeding on *C*Las-infected citrus plants may be due to the fact that *C*Las infection could destroy the midgut epithelial cells of the ACP nymphs, so causing them to be unable to fully absorb and utilize nutrients. These unabsorbed nutrients are then excreted from the body in the form of honeydew. In our study, we found that *C*Las infection clearly reduced the proportion of Ser, Trp, and Ala in the honeydew of ACP nymphs. Thompson (2006) reported that the absorption of Ser, Ala, Glu, and Arg in host plants plays an important role in the survival rate of *B. tabaci*. Therefore, we speculated that the reason for the reduced survival rate of the nymphs may be related to Ser, Trp, and Ala absorption by the ACP nymphs not being fully utilized.

## Conclusion

Our study has revealed that *C*Las infection alters the amino acid contents in the phloem sap of citrus plants, in ACP adults, and in the honeydew produced. *C*Las infection decreased the total amount of amino acids and the proportion of EAAs in host plants and also increased the proportion of EAAs in ACP but affected the utilization of EAAs by ACP. This information expands our knowledge concerning the nutritional requirements for plant phloem-limited pathogens and their insect vectors. *C*Las infection significantly affects the contents, proportions, and utilization efficiency of different amino acids in citrus plants and ACP nymphs, which together lead to a developmental pattern of ACP, which is more conducive to *C*Las transmission. The research results that were previously published by our laboratory in which ACP fed on citrus plants infected with HLB bacteria improved the development duration and fecundity of ACP, which supports the results of this study (Ren et al., 2016). The physiological mechanisms in relation to the importance of amino acids, nutritional requirements, and energy transformation are areas that still require further investigation.

## Data Availability Statement

The raw data supporting the conclusions of this article will be made available by the authors, without undue reservation.

## Author Contributions

L-HZ, S-LR, and B-LQ: conceived and designed the experiments. L-HZ, S-LR, Z-QS, P-PX, DO, and L-JW: performed the experiments. L-HZ, S-LR, and WS: analyzed the data. WS and B-LQ: contributed to reagents, materials, and analysis tools. L-HZ and B-LQ: wrote the manuscript. All authors contributed to the article and approved the submitted version.

## Conflict of Interest

The authors declare that the research was conducted in the absence of any commercial or financial relationships that could be construed as a potential conflict of interest.

## Publisher’s Note

All claims expressed in this article are solely those of the authors and do not necessarily represent those of their affiliated organizations, or those of the publisher, the editors and the reviewers. Any product that may be evaluated in this article, or claim that may be made by its manufacturer, is not guaranteed or endorsed by the publisher.
